# Intended cost reduction in laparoscopic appendectomy by introducing the endoloop: a single center experience

**DOI:** 10.1186/s12893-017-0277-z

**Published:** 2017-07-11

**Authors:** Matthias Mehdorn, Olaf Schürmann, H. Maximilian Mehdorn, Ines Gockel

**Affiliations:** 1 Department of Visceral, Transplant, Thoracic and Vascular Surgery, University Hospital Leipzig, Liebigstraße 20, 04103 Leipzig, Germany; 2 Department of Operative Medicine, commercial managements, University Hospital Leipzig, Liebigstraße 20, 04103 Leipzig, Germany; 3 Mehdorn Consilium, Prüner Gang 7, 24103 Kiel, Germany; 4 Department of Surgery, Clinic for Visceral, Transplant, Thoracic and Vascular Surgery, UKL, University Hospital of Leipzig, Liebig Strasse 20, 04103 Leipzig, Germany

**Keywords:** Laparoscopic appendectomy, Endoloop, Stapling device, Cost-reduction

## Abstract

**Background:**

Cost reduction measures in medicine are gaining greater importance nowadays, especially in high-volume procedures such as laparoscopic appendectomy (LAE). Currently there are two common methods of dissecting the appendix from the caecal pole: linear stapler and endoloops. The endoloop is the cheaper device but can only be used in uncomplicated cases of appendicitis. Therefore both methods are used in LAE depending on intraoperative findings. The goal of this study was to retrospectively evaluate possible cost reduction due to increased use of endoloop in LAE in our general surgery department of a tertiary referral university hospital.

**Methods:**

We previously used the stapler for appendix dissection in LAE as our local protocol but introduced the endoloop as standard method in 2015 to reduce intraoperative costs. We conducted a retrospective analysis of patients who underwent LAE between June 2014 and October 2015 in our department. Our purpose is to show the effects on cost reduction during the introductory period adjusting for a potential bias due to the individual learning curve of every surgeon. We estimated costs for LAE by taking into account average device costs and duration of operation (DO) as well as patient outcome.

**Results:**

A total of 177 patients underwent LAE, 73 in 2014 (phase I) and 104 in 2015 (phase II). The median DO was 61 (± 24 SD) min during the entire period, and increased by 14 min from phase I to II (from 51 (±23 SD) min to 65 (±24 SD) min respectively, *p* < 0.001). The use of endoloops increased from 10% to 55% (*p* < 0.001). Patients’ characteristics and outcomes did not differ significantly. A median saving of 5.9€ per operation was calculated in phase II compared to phase I (*p* = 0.80).

**Conclusion:**

Introducing the endoloop as standard device for LAE leads to a marginal reduction in intraoperative costs without increasing negative outcomes. In our model the cost-reduction achieved by cheaper devices was overcome by increased costs for DO during the initial phase of use of endoloops. A longer follow up might show a more pronounced cost reduction.

## Background

Acute appendicitis has an incidence of 100/100,000 per year in Western Europe, of which about 20% have already perforated when admitted to hospital [1]. Current standard therapy is the urgent laparoscopic appendectomy (LAE) [2, 3] with about 140,000 appendectomies performed in Germany in 2015 [4].

Separating the appendix from the caecum can be achieved using different devices: The most common one is the linear stapler because it can also be used in more advanced stages of appendicitis, e.g. with inflammatory infiltration of the caecal pole. Alternatively, ligation of the appendix can be performed using an endoloop with absorbable sutures. Both methods have been proven efficient and safe [5–7] although recent publications suggest that using the endoloop might be associated with a slightly higher rate of stump insufficency [8, 9].

The application of an endoloop requires a more thorough preparation of the appendix and its mesoappendix and utilising an endoloop is more difficult than a stapler [5, 6]. Furthermore, LAE is one of the first intraabdominal operations performed by residents in training. The combination of those two factors might increase duration of operation (DO) compared to stapler procedures.

The German DRG (Diagnosis Related Groups) system reimburses LAE with a standardised amount irrespective of the method used for dissecting and resecting the appendix. The reimbursement is calculated by the average costs and average length of stay (LOS) in German index hospitals. With this system in place a patient has to be discharged at a lower to mean LOS in order to be profitable for the hospital. In LAE the target LOS is between the 2nd and 4th post-operative day. Besides an early discharge date, faster surgery using cheaper instruments in theatres is another way to increase profit. One stapler including one charge of staples costs about 280€, whereas using endoloops results in costs of about 45€ per usage. This results in a cost difference of about 235€. By using other disposable devices like the Harmonic Scalpel or LigaSure costs may increase but DO is reduced [10].

The timing of an individual surgery is crucial as it should weigh the benefit of early surgery against possible drawbacks of night time surgery and its subsequent influence on costs [11–15].

The purpose of this retrospective study was to analyse the extent to which it is possible to use the endoloop in LAE as a standard device in regards to the different stages of appendicitis. We furthermore wanted to analyse if the introduction of the endoloop for LAE as standard device is appropriate to reduce intraoperative costs. As main budget factors we considered material costs and costs for DO in order to calculate an approximate overall cost for comparison of endoloop versus stapler.

## Methods

At our university tertiary referral teaching hospital, we introduced the endoloop as standard technique by January 2015 with the intention to reduce intraoperative costs. A retrospective analysis of all patients receiving LAE between June 2014 and October 2015 was carried out with June to December 2014 being phase I and January to October 2015 representing phase II. To analyse the immediate effects of introducing the endoloop as standard device we included a limited number of patients after its introduction in phase I and matched the group size with an approximately equal number of patients from the period prior to the introduction of the endoloop. We chose this approach accepting a degree of bias due to the individual learning curve of every surgeon to evaluate possible immediate effects of the new standard technique.

Data on age, sex, method of resection (stapler or endoloop), histological findings, DO, post-operative LOS, as well as post-operative complications before and after introduction of endoloop were collected. Additionally, we determined surgeons’ experience by using our respective group of duty as a surrogate, i.e. young (1st to 4th year)/old residents (~5th to 6th year), specialists and consultants. Furthermore, the time of day the operation took place (day, late and night shift) was recorded to evaluate its influence on DO.

We use a three trocar approach with multi-use trocars and standardised sutures. In phase I the stapler was the standard approach; in Phase II the endoloop was the standard device but if an advanced appendicitis with inflammation of the proximal appendix, the caecal pole or perforation was encountered, resection was performed with the stapler. For skeletonization and preparation of the appendix monopolar cautery is used. It is up to the operating surgeon’s discretion which device to use according to the criteria mentioned above. Once intraoperative decision on method of resection is made the respective device is opened. From the operating notes it is not possible to conclude if redundant endoloops or stapler firings were used and we therefore performed calculations assuming the reported number was opened and then used.

We use the 35 mm ETS Articulating Linear Cutter (Ethicon Endo Surgery, Norderstedt, Germany) which costs about 280€ with one set of staples, an extra staple set costs about 160€. The endoloop is an absorbable PDS II, suture strength 0 endoloop (Ethicon Endo Surgery) of which 3 loops are required (2 proximal loops, one distal), resulting in costs of about 45€ per case. One minute of operating room (OR) time cost about 12€ in our institution in 2015. This is a mean calculated for day and night time, including costs for anesthesia and room cleaning and standard materials such as sutures or drapings. From those figures the average cost of a procedure was calculated by adding costs of OR time and material costs.

We did not intend to perform a cost-consequence analysis or a cost-effectiveness analysis as those models do not seem appropriate to us for this certain setting.

Follow-up conducted was limited to the post-operative hospital stay as further routine follow-ups are performed by a patient’s local general practitioner. We therefore only included in-hospital complications or complications registered by readmission to our department.

Patient data was collected from electronic patient charts, assembled in Excel 2010, version 14.5.1 (Microsoft Corporation, WA, USA) and analysed in SPSS 20 (IBM Statistics, Chircago, Ill., USA). For all continuous variables, the median and standard deviation was calculated and therefore Mann-Whitney-U test (MWU test), Kruskal-Wallis test or X^2^ test were used. As costs showed a standard distrubution we used the unpaired t-test. A multivariate analysis was performed to determine to which extent DO is influenced by method of dissection, surgeon’s experience, time ofn day and patient age. *P*-values were set at 0.05 and graphs were created using SPSS 20.

## Results

One hundred seventy seven patients underwent LAE between June 2014 and October 2015. Demographics and histological findings are listed in Table 1a and b. Table 2 displays procedure-specific data over the course of time.

**Table 1:** Descriptive demographics (a) and histological findings (b) of study collective. Testing of X^2^ or Mann-Whitney-U-(MWU)-test did not reveal any significant difference between phase I and II for all categories listed below

	Total	Phase I	Phase II	*P*-values
a) Demographics				
No. of procedures	177	71	106	
Male patients, absolute number (relative value)	77 (43.5%)	27 (38%)	56 (52.8%)	0.28
Female patients, absolute number (relative value)	100 (56.5%)	44 (62%)	50 (47.2%)	
Age, years (±SD)	27.8 (±15.5) y	26.4 (±13.5) y	29.4 (±16.7) y	0.29
LOS, days (±SD)	3 (±5.3) d	3 (±1.7) d	3 (±6.3) d	0.38
b) Histology				
No appendicitis	9 (5.1%)	1 (1.4%)	8 (7.7%)	0.16
Minimal Appendicitis	31 (20.9%)	17 (23.3%)	20 (19.2%)	
Ulcero-Phlegmonous appendicitis	47 (26.6%)	24 (32.9%)	23 (22.1%)	
Highly active ulcero-phlegmonous appendicitis	66 (37.3)	26 (35.9%)	40 (38.5%)	
Ulcero-phlegmonous appendicitis with perforation	17 (9.6%)	5 (6.8%)	12 (11.5%)

**Table 2:** Procedure specific data showing a significant increase of use of endoloop in phase II, concurrent with a significant increase in DO

	Total	Phase I	Phase II	*P*-values
Endoloop, absolute number (relative value)	65 (36.7%)	7 (9.9%)	58 (54.7%)	<0.001
Stapler, absolute number (relative value)	112 (63.3%)	64 (90.1%)	48 (45.3%)	
DO in min (±SD)	61 (±24) min	51 (±23) min	65 (±24) min	<0.001
DO Endoloop in min (±SD)		49 (±5) min	55 (±18.6) min	0.081
DO stapler in min (±SD)		54.5 (±24.7) min	71 (±25.2) min	<0.001

### Clinical results

A significant difference could be found for DO: 51 min (±23 min SD) in phase I vs. 65 min (±24 min SD) in phase II (*P* < 0.001); the use of endoloops increased significantly in phase II (9.9% vs 54.7% of all LAE respectively; *P* < 0.001).

In both phases, no significant differences were noted with regard to patients’ age (*P* = 0.29), sex (*P* = 0.28), histologic findings (Table 1), surgeons’ experience (*P* = 0.27) and post-operative LOS (*P* = 0.38). In phase I there was a trend towards shorter DO with increasing experience which was not found in phase II. None of those were statistically significant with *p* = 0.82 and *p* = 0.23 for phase I and II respectively. No significant difference was observed for the time of day at which the operation was performed (*P* = 0.61). The X^2^-test could not demonstrate a significant difference between which group of surgeons preferred a certain method, endoloop or stapler (*P* = 0.13).

**Fig. 1:**
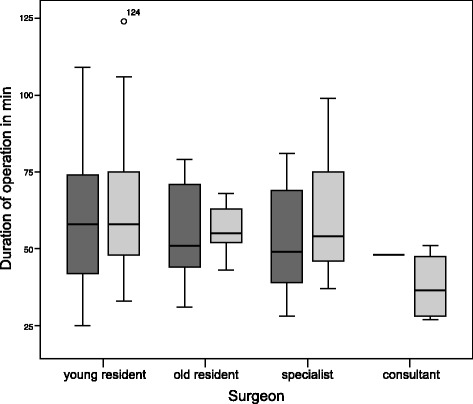
Duration of operation stratified by surgeon’s experience and time period; *Dark grey* displaying phase I; *light grey* displaying phase II

**Fig. 2:**
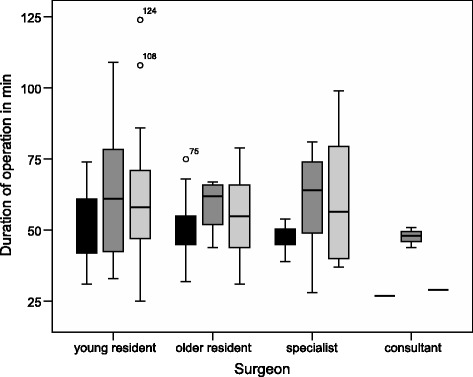
Median duration of operation stratified by shift and surgeon. Day shift from 7 a.m. to 4 p.m., *black* boxes; late shift from 4 p.m. to 12 p.m., *dark grey* boxes; night shift from 12 p.m. to 7 a.m., *light grey* boxes

The increase of DO from phase I to phase II is evident regardless of the experience (Table 3, Fig. 1). DO seems to increase at later hours of the day, but no statistically significant difference was observed, neither with regards to phase nor group of surgical expertise: young residents *P* = 0.68, older residents *P* = 0.72, specialists *P* = 0.39 and consultants *P* = 0.36 (Fig. 2).

**Table 3:** DO by surgical experience. MWU-test showed significant differences for older residents and specialists

	Total	Phase I	Phase II	*P*-values comparing both phases
Young resident, DO in min (±SD)	61 (±19.2) min	54 (±20.2) min	63,5 (±18) min	0.10
Older resident, DO in min (±SD)	58 (±28.5) min	51 (±33.2) min	65 (±24.7) min	0.039
Specialist, DO in min (±SD)	66.5 (±27.2) min	49 (±17) min	75 (±28.5) min	0.009
Consultant, DO in min (±SD)	49.5 (±24.3) min	48 min	51 (±26.1) min	n.a.; only one case in phase I
*P*-values according to each phase	0.56	0.82	0.23	

Young residents show a tendency towards longer duration of operation

Surgical complications were distributed equally for both phases (2 in phase I and 3 in phase II, *p* = 1.00), ranging from superficial wound site infection in one patient on immunosuppressants after liver transplantation to persistent intraabominal abscess due to stumpf insufficiency after stapler appendectomy that required reintervention. We could not identify a device specific increase in complications in phase II. In both groups about one third of histologically or macroscopically perforated appendicitides caused intraabdominal abscess formation. In phase II also 3 histologically intact but highly active ulcero-phlegmonous appendicitides caused perityphlitic abscesses. Perityphlitic abscesses were treated with postoperative abdominal lavage via intra-operatively placed drainage catheters and intravenous antibiotics. One patient died due to severe sepsis at presentation to our emergency department which did not improve despite resecting the necrotic appendix. Another patient suffered from severe sepsis post-operatively due to a perforated necrotic appendicitis but consequently fully recovered.

Table 4 sums up characteristics of both subgroups in phase II, the stapler and the endoloop cohort.

**Table 4:** Appendectomy in phase II by method of dissection with regard to median age, duration of operation and postoperative length of stay

Phase II	Age, median (±SD) in years	DO, median (SD±) in min	LOS, median (±SD) in days
Stapler,	39.5 (19.8) y	72.5 (19.8) min	3 (9.8) d
Endoloop	26.3 (9.9) y	55 (18.6) min	2 (0.9) d
*P*-Values	*P* < 0.000	*P* < 0,001	*P* < 0.001

### Economic considerations

With regards to possible savings, we suggested to save a maximum amount of 235€ (=price difference of instruments) per patient when endoloops are used instead of the linear stapler presuming that in every case the endoloop could be used. However, the DO is another crucial factor influencing the total surgical costs of LAE. Considering both those factors we calculate average costs per procedure by adding costs per device and per time in the OR. The average costs per phase and device are summarised in Table 5.

**Table 5:** Calculation of average costs per procedure according to phase and device

	Average DO in min	DO costs	Material costs	Costs per procedure	*P*-values for “Cost per procedure”
Phase I					
Endoloop	49	588	45€	633€	<0.001
Stapler	54.5	654	280€	934€	
Difference Endoloop vs stapler				301€	
Phase II					
Endoloop	55	660	45€	705€	<0.001
Stapler	71	852	280€	1.132€	
Difference Endoloop vs stapler				427 €	

Price per minute OR time is 12€

Since there was no exclusive use of either device in phase I and II as surgical indications vary as described above we calculated the average total cost difference for all procedures performed in the two phases which resulted in 5,90€ (*p* = 0.80) while material costs decreased by 105.4€ (*p* < 0.001). Table 6 sums up the important figures comparing both phases.

**Table 6:** Calculation of average cost per procedure and average cost per device stratified by phase

	No of Procedures with endoloop	Price per procedure with endoloop	No of procedures with stapler	Price per procedure with stapler	Average cost per device	Average cost per procedure
Phase I	7	633€	64	934€	256.8€	904.3 €
Phase II	58	705€	48	1.132€	151.4€	898.4 €
*P*-values	<0.001	<0.001	<0.001	<0.001	<0.001	0.80

The *p*-values are indicated to the right or below of their respective row or column

## Discussion

We sought to evaluate the feasability of introducing the endoloop as standard resection device for LAE and its possible subsequent intraoperative cost-reduction for our tertiary referral teaching hospital.

Calculating intraoperative costs for LAE consists of two factors: i) costs for DO and ii) average material costs for resection devices. Therefore minimising both of them will result in the lowest intraoperative costs possible.

### Duration of operation (DO)

We found an increase in DO after introduction of the endoloop (from 51 ± 23 min SD in phase I to 65 ± 24 min SD in phase II). This increase is also present when analysing the surgeons’ sub-groups although the correlation is not as pronounced in phase II compared to phase I. DO in laparoscopic appendectomy varies considerably in literature independant of the device used. No reports exist so far how operative experience influences DO. DOs between 50 and 62 min have been described for stapling [5, 16]; for the endoloop a range from 47 min up to 75.4 min is reported [6, 9]. From those results one might conclude that stapling is faster. But as time spans of DO cover a wide range regardless of the device it remains elusive which one might be most suitable to reduce DO. As endoloop and stapler cannot be used interchangeably but have to be used according to the severtitiy of appendicitis they cannot be compared independetly in all cases. This would not reflect clinical reality which is the reason why our approach was to calculate the average values of both methods and their respective costs.

The increase of DO in phase II may be a temporary phenomenon: the introduction of endoloop represented a new technique even for many of our specialists. A learning curve was therefore expected as in our personal experience performing roughly 3 cases with endoloops was required to learn proper handling and placement of the loop. The endoloop procedures performed in phase I were exclusively performed by one specialist who had previously worked with this device in another hospital. Therefore these DOs are not representative or comparable to those in phase II. The other surgeons had never used endoloops before. Further follow-up in this regard is mandatory and may elucidate the bias by learning the new technique.

In our study DO does not vary over the course of the day. There is a tendency towards an increase in DO towards later hours (Fig. 2) which is in accordance with Yaghoubian et al. [14]. In contrast, incidence of complications do not differ throughout the course of the day in appendectomies [14]. As a delay of surgery from the onset of symptoms appears to influence DO and post-operative complications surgical procedures are routinely performed irrespective of the time of day [11–13, 15].

### Costs and selective use of devices

The second variable in our study is the intended reduction of intraoperative costs for devices. The more frequent use of endoloops, rising from 10% in phase 1 up to 55% in phase II meant that the remaining difficult cases were reserved for the use of the stapler device. This is reflected by the longer DO and LOS in the stapler subgroup of phase II (Table 4). The intention to use the endoloop and therefore preparation is commenced with monopolar cautery and scissors instead of using two stapler firings for preparation and resection might have led to an increase in DO as well. Whilst a cost reduction of material costs by 105€ per procedure was achieved (Table 6) it was opposed by an increase in DO of 14 min. Our policy of routinely using the stapler in addition to the endoloop only in more complicated cases is similar to Sahm et al. [9]. They were able to utilise the endoloop in 97.3% of all LAE. Those numbers would lead to an average material cost of about 52€ per case in contrast to 151€ in our study. We utilised the endoloop in only 55% of all cases. Reasons for that might be a surgeon’s hesitance to perform a surgery with an unfamiliar equipment and technique which resulted in the surgeon’s choice to only use the endoloop in very early stages of appendicitis. Therefore maximising the use of endoloop with growing experience in one institution should become a factor in reducing costs. This seems very feasable when considering the data presented by Sahm et al. which shows promise to widen the application of endoloops to even more advanced cases. Furthermore, refraining from using single-use trocars or single-use instruments for appendix skeletonization and instead using multi-use trocars and monopolar cautery for preparation of the appendix, as is the case in our institution, has proven to be the most cost-efficient way [10, 17]. This is in keeping with the recommendations of the WSES for LAE [18]. There are also other approaches to minimise costs in LAE such as single port laparoscopy using a surgical glove [19]. This method provides cost reduction compared to conventional SILS-LAE. But we would not expect monetary benefits in comparision with our standard set-up of multi-use trocars.

A faster recovery and earlier discharge have been demonstrated in LAE compared to open surgery [20]. No difference in overall LOS did occur after introduction of the endoloop in our cohort. In comparison to the literature, our patients had a shorter post-operative LOS [5, 21], which might be led by the DRG-system that expects a mean LOS of 4 days. Ultimately however the point of time for discharge is a clinical decision which requires decreasing inflammatory parameters, normal bowel function, bearable postoperative pain and regular wound healing.

By using the aforementioned approach we calculated intraoperative savings of 5.9€ per LAE in 2015 compared to 2014 (Table 6). When calculating and comparing intraoperative costs most studies only consider costs of devices [6, 10, 17]. Only Beldi et al. [5] took costs of operating time into account and calculated the difference between material and OR-costs. The basis of their data is a nationwide register and not a single institution hence a heterogenous pool of surgeons and surgical settings is compared. They calculated a difference of 248€ per case with use of stapler compared to endoloop. This difference is mainly driven by costs for devices as they described a difference in DO of about 2 min in favor of the stapling device. We found an even more pronounced differences - about 427€ - when comparing endoloop and stapler LAEs in phase I. Another study compared open to laparoscopic appendectomy with endoloops as laparoscopic surgery in general is more expensive due to material costs [22]. In that study operative costs for LAE were set to be a total of 148€ without specifying exactly the calculation of operative costs. Lukish et al. chose a similar approach in comparing costs of pediatric laparoscopic appendectomy with eiter Harmonic Scalpel and endoloop or two firings of stapler and found a financial advantage of 572$ in favor of stapling [23]. This difference was mainly driven by a longer DO of about 14.9 min and costs for use of Harmonic Scalpel for the mesoappendix when using the endoloop. We had a similar increase in DO but were able to reduce costs by using monopolar cautery instead of the single use Harmonic Scalpel. Suprisingly we could only demonstrate a cost-saving of 5.9€ per procedure in favour of endoloop during phase II when calculating the average costs for LAEs. The main reasons for that comparatively smaller cost-reduction are i) the increase in DO and ii) the persistantly high use of stapling devices.

Considering all patient characteristics, intraoperative and histological findings as well as surgeon’s experience did not differ significantly the only variable that explains the pronounced increase in DO is the difference in device used. The increase in DO almost negates the financial benefits of using the cheaper device. As complications and post-operative LOS did not differ between both phases we suggest that consequently intraoperative costs are the biggest variable contributing to the total costs in a case of acute appendicitis. The cost calculations presented herein are only rough estimations and only provide a trend as costs are not routinely monitored per patient in the hospital (e.g. Day Mix Index) and costs of devices and time in the OR may vary significantly between hospitals and countries. Additionally our data represents the introductory period of the new technique thus the results might be influenced by the surgeons’ inexperience that leads to a relatively greater increase in duration of operation. To eliminate this effect a much longer study period is needed. As our intention was to show direct effects on costs we only chose the limited time frame.

All these limitations of our study taken into consideration: We are the first to report the direct effects on intraoperative costs upon introducing the endoloop as standard method for resecting the appendix in LAE. Our results may be biased by every surgeon’s inexperience with the endoloop in the beginning and in relation to surgeons’ individual learning curves. We propose a training duration of about three cases to be able to place the endoloop without any technical difficulties. A longer prospective follow-up period is required to fully evaluate the influence of the learning curve and the steady state once the technique has been mastered to possibly demonstrate greater advantages of the endoloop. Additionaly limiting the patient inclusion criteria to early to mild appendicitis with randomised use of either endoloop or stapler could elucidate financial benefits but would then not reflect clinical reality.

## Conclusion

Although the endoloop is a practical and cheap device for resecting the appendix in laparoscopic appendectomy, the “true” economic benefit of the endoloop as standard method for LAE is not as high as expected a priori when regarding costs of each device. In our series, costs by prolonged DO negated some of the expected savings by endoloop for various possible reasons, e.g. surgeon’s learning curves, surgeons at an early stage of training. Growing experience with the endoloop might reveal more pronounced cost-reduction possibilities.

## Abbreviations

DO: Duration of operation
DRG: Diagnosis related group
LAE: Laparoscopic appendectomy
LOS: Length of stay
MWU: Mann-Whitney-U-Test
OR: Operating room
SD: Standard deviation
SILS: Single incision laparoscopic surgery
WSES: World society of emergency surgeons
